# Germline mutation in the *RAD51B* gene confers predisposition to breast cancer

**DOI:** 10.1186/1471-2407-13-484

**Published:** 2013-10-19

**Authors:** Lisa Golmard, Virginie Caux-Moncoutier, Grégoire Davy, Essam Al Ageeli, Brigitte Poirot, Carole Tirapo, Dorothée Michaux, Catherine Barbaroux, Catherine Dubois d’Enghien, André Nicolas, Laurent Castéra, Xavier Sastre-Garau, Marc-Henri Stern, Claude Houdayer, Dominique Stoppa-Lyonnet

**Affiliations:** 1 Institut Curie, Department of Tumour Biology, 26 rue d’Ulm, 75248, Paris, Cedex 05, France; 2 Institut Curie, INSERM U830, 26 rue d’Ulm, 75248, Paris, Cedex 05, France; 3 Université Paris Descartes, Sorbonne Paris Cité, 12 rue de l’école de Médecine, 75270 Paris, Cedex 06, France

**Keywords:** Genetic predisposition, Breast cancer, Ovarian cancer, *RAD51* paralogs, *RAD51B*

## Abstract

**Background:**

Most currently known breast cancer predisposition genes play a role in DNA repair by homologous recombination. Recent studies conducted on *RAD51* paralogs, involved in the same DNA repair pathway, have identified rare germline mutations conferring breast and/or ovarian cancer predisposition in the *RAD51C, RAD51D* and *XRCC2* genes. The present study analysed the five *RAD51* paralogs (*RAD51B, RAD51C, RAD51D, XRCC2, XRCC3*) to estimate their contribution to breast and ovarian cancer predisposition.

**Methods:**

The study was conducted on 142 unrelated patients with breast and/or ovarian cancer either with early onset or with a breast/ovarian cancer family history. Patients were referred to a French family cancer clinic and had been previously tested negative for a *BRCA1/2* mutation. Coding sequences of the five genes were analysed by EMMA (Enhanced Mismatch Mutation Analysis). Detected variants were characterized by Sanger sequencing analysis.

**Results:**

Three splicing mutations and two likely deleterious missense variants were identified: *RAD51B* c.452 + 3A > G, *RAD51C* c.706-2A > G, *RAD51C* c.1026 + 5_1026 + 7del, *RAD51B* c.475C > T/p.Arg159Cys and *XRCC3* c.448C > T/p.Arg150Cys. No *RAD51D* and *XRCC2* gene mutations were detected. These mutations and variants were detected in families with both breast and ovarian cancers, except for the *RAD51B* c.475C > T/p.Arg159Cys variant that occurred in a family with 3 breast cancer cases.

**Conclusions:**

This study identified the first *RAD51B* mutation in a breast and ovarian cancer family and is the first report of *XRCC3* mutation analysis in breast and ovarian cancer. It confirms that *RAD51* paralog mutations confer breast and ovarian cancer predisposition and are rare events. In view of the low frequency of *RAD51* paralog mutations, international collaboration of family cancer clinics will be required to more accurately estimate their penetrance and establish clinical guidelines in carrier individuals.

## Background

Breast cancer is currently the most common cancer and the leading cause of cancer deaths in women worldwide [1]. Abnormal familial aggregations fitting a model of autosomal dominant breast cancer genetic predisposition account for about 5% of cases [2]. *BRCA1* and *BRCA2* are the two major genes, but explain only about 20% of inherited breast cancers [3-5]. About ten genes are known to be involved in breast cancer predisposition, either isolated or associated with other cancers, with variable breast cancer risks. Approximately 50% of familial breast cancers remain unresolved by any of these genes after genetic testing [6].

Most currently known breast cancer predisposition genes play a role in the repair of DNA double-strand breaks by homologous recombination: *BRCA1* and *BRCA2*, associated with a high risk of breast cancer, and *BRIP1* and *PALB2*, associated with a moderate risk [7-9]. While breast cancer predisposition is conferred by mono-allelic germline mutations in these genes, bi-allelic germline mutations in *BRCA2*, *BRIP1* and *PALB2* result in Fanconi anaemia, an autosomal recessive inherited syndrome characterized by multiple developmental abnormalities and predisposition to various cancers [10-12].

Genetic studies were recently conducted on the *RAD51* paralogs, involved in the same DNA repair pathway: BRCA2 protein loads RAD51 monomers at DNA double-strand break sites; RAD51 recruitment also depends on the RAD51 paralog family [13]. Bi-allelic mutations resulting in Fanconi anaemia were identified in *RAD51C* and *XRCC2*[14,15]. Mono-allelic germline mutations conferring breast and ovarian cancer predisposition were identified in *RAD51C* and *RAD51D*[16,17]. *XRCC2* mutations were then detected in breast cancer families but a subsequent population-based study failed to confirm an association between *XRCC2* variants and breast cancer risk [18,19]. Johnson et al. conducted a study on *RAD51B* in breast cancer families and did not detect any mutations [20]. The *XRCC3* gene has not yet been studied.

In this study, we analysed the five *RAD51* paralogs (*RAD51B, RAD51C, RAD51D*, *XRCC2, XRCC3*) in 142 unrelated patients with breast and/or ovarian cancer to estimate their contribution to breast and ovarian cancer predisposition.

## Methods

### Patients

The study was conducted on 142 unrelated patients with breast and/or ovarian cancer either with early onset or with a breast/ovarian cancer family history. Patients had been previously tested negative for a *BRCA1/2* mutation, selected either for a predisposition probability higher than 70% according to the Claus model [2] or for enrichment in ovarian cancer cases: 87 patients (61%) had a personal or family history of both breast and ovarian cancer, 10 patients (7%) had a personal or family history of ovarian cancer only and 45 patients (32%) had a personal or family history of breast cancer only (Table 1). All patients attended a visit with a geneticist and a genetic counsellor in a family cancer clinic, mostly at the Institut Curie, Paris, France. Patients gave their informed consent for genetic testing. The study was approved by the local Ethics Committee in Institut Curie.

**Table 1:** Patient personal and family history of breast/ovarian cancer

**Personal and family history**	**n (%)**
**Personal history**	
Ovarian adenocarcinoma before the age of 70	4 (3)
Breast adenocarcinoma and ovarian cancer	6 (4)
Breast adenocarcinoma before the age of 36	1 (1)
Family history*	
2 ovarian cancer cases	81 (57)
2 breast cancer cases	36 (25)
3 breast cancer cases	8 (6)
1 breast cancer case and 1 ovarian cancer case	6 (4)

*Family history was defined in first- or second-degree relatives in the same lineage.

### Genomic DNA analysis

Genomic DNA was extracted from 2 mL whole-blood samples collected on EDTA with the Quickgene 610-L automated system (Fujifilm) according to the manufacturer’s instructions. *RAD51* paralog mutation screening was performed on coding exons and exon-intron junctions by multiplex PCR and Enhanced Mismatch Mutation Analysis (EMMA) [21] except for 2 *RAD51B* exons which were analysed by simplex PCR and direct sequencing (Additional file 1: Table S1 and Additional file 2: Table S2). PCR products showing abnormal EMMA profiles were analysed by sequencing on an ABI PRISM 3130XL Genetic analyzer (Applied Biosystems).

### mRNA analysis for *RAD51C* splicing mutations

RNA was extracted from lymphoblastoid cell lines using TRIzol reagent according to the manufacturer’s instructions (Invitrogen). 2 μg of total RNA from each sample was used for reverse transcription in a 40 μL reaction using the GeneAmp RNA PCR Core kit according to the manufacturer’s instructions (Applied Biosystems). cDNA was amplified with forward and reverse primers gcattcagcaccttcagctt and ctttcggtcccaatgaaaga for *RAD51C* exon 5 skipping, tgacctgtctcttcgtactcg and for *RAD51C* exon 8 skipping.

### RAD51B immunohistochemistry

For RAD51B immunostaining, 4-μm-thick paraffin sections were cut and mounted on glass slides (Superfrost+, Menzel Glazer). Preparations were dried for one hour at 58°C, then overnight at 37°C. Sections were deparaffined with toluene and rehydrated with ethanol. Preparations were pretreated with citrate buffer (0.01 M citric acid pH 6.0), and a heat-based antigen retrieval method was used prior to incubations. Endogenous peroxidase was blocked using 3% hydrogen peroxidase solution for 5 minutes. The primary anti-RAD51B antibody used (clone NBP1-66539, dilution 1/200) was from Novus Biologicals. Sections were incubated for 15 minutes at 22°C with the primary antibody followed by staining with anti-rabbit HRP antibody (Leica Biosystems) for 10 minutes. Sections were then revealed in a diaminobenzidine solution for 15 minutes and stained with hematoxylin for 7 minutes.

### Missense variants pathogenic prediction

Three bioinformatics tools were used for missense variants pathogenic prediction: Align-GVGD [22,23], SIFT [24,25] and Polyphen-2 [26,27]. Multiple sequence alignment (MSA) for Align-GVGD and SIFT analysis was an alignment of protein sequences of 11 species: Human (*Homo sapiens*), Chimpanzee (*Pan troglodytes*), Macaque (*Macaca mulatta*), Mouse (*Mus musculus*), Rabbit (*Oryctolagus cuniculus*), Dog (*Canis familiaris*), Cat (*Felis catus*), Bovine (*Bos taurus*), Opossum (*Monodelphis domestica*), Platypus (*Ornithorynchus anatinus*), Chicken (*Gallus gallus*) and Frog (*Xenopus tropicalis*). Missense variants were interpreted as likely deleterious if they were classified as deleterious or probably damaging by the three tools.

### Statistical analysis

Frequencies of mutations and likely deleterious variants were compared between the cases and two control samples from online databases: European-American controls from Exome Variant Server [28] and European controls from 1000 Genomes project [29]. In a first step the two control sample variant frequencies were compared using Fisher’s exact test in order to check there was no significant difference. In a second step the control samples were pooled and the overall control variant frequency was compared with the case sample one, using Fisher’s exact test. All the tests were two-sided, with a p-value of 0.05 considered significant. Computations were performed using the XLSTAT-2013 software.

## Results

Three splicing mutations and two likely deleterious missense variants were identified in these 142 patients: *RAD51B* c.452 + 3A > G, *RAD51C* c.706-2A > G, *RAD51C* c.1026 + 5_1026 + 7del, *RAD51B* c.475C > T/p.Arg159Cys and *XRCC3* c.448C > T/p.Arg150Cys (Table 2). No mutation was detected in the *RAD51D* and *XRCC2* genes (See Additional file 3: Table S3 for all variants and polymorphisms detected in this study).

**Table 2:** Mutations and likely deleterious variants, their effect on splicing or protein, cancer history of carriers

**Gene**	**Genetic variation**	**Variant class**	**Effect on splicing**	**Predicted effect on protein (Align-GVGD class**^**†**^**)**	**Personal cancer history (age at diagnosis)**	**Family cancer history (age at diagnosis)**	**Controls**^**‡**^
*RAD51B*	c.452 + 3A > G	Splicing mutation	Exon 5 skipping by *in silico* prediction*	Unstable or truncated protein, confirmed by negative IHC	BC (34)	Paternal aunt, BC (58); 3^rd^ degree relative, OC (29)	-
*RAD51B*	c.475C > T, p. Arg159Cys	Likely deleterious missense variant	No predicted effect	ATP-binding domain, highly conserved amino acid, Grantham 180 (Class C65)	BC (54)	Sister, BC (45); Sister’s daughter BC (45)	2/4299
*RAD51C*	c.706-2A > G	Splicing mutation	Exon 5 skipping confirmed by mRNA analysis	44 amino acids loss in ATP-binding domain	BC (39)	Paternal aunt^§^, OC (67)	-
*RAD51C*	c.1026 + 5_1026 + 7del	Splicing mutation	Exon 8 skipping confirmed by mRNA analysis	Unstable or truncated protein	BC (38), OC (51)	Father, PC (69); Paternal grandmother, UC (66); Paternal grandfather, SC (69)	-
*XRCC3*	c.448C > T, p. Arg150Cys	Likely deleterious missense variant	No predicted effect	ATP-binding domain, highly conserved amino acid, Grantham 180 (Class C65)	BC (63)	Mother, OC (61); Maternal aunt, BC (55); Maternal aunt, BC (73); Maternal aunt, BC (76); Maternal aunt, BC (63, 79)	1/4276

BC: Breast cancer, OC: Ovarian cancer, PC: Pancreas cancer, UC: Uterine cancer, SC: Stomach cancer.

*See text for details.

†Align-Grantham Variation Grantham Deviation (Align-GVGD) classes range from C0 to C65; C65 class variants are the most likely to interfere with protein function [22,23].

§This paternal aunt also carried the *RAD51C* c.706-2A > G mutation.

‡Frequency in controls in online databases: Exome Variant Server [28], dbSNP [38], 1000 Genomes [29]. The three splicing mutations have never been described. The 2 missense variants were reported only in Exome Variant Server, in European-American populations. Their frequencies in European-American populations are reported in this table.

**Figure 1:**
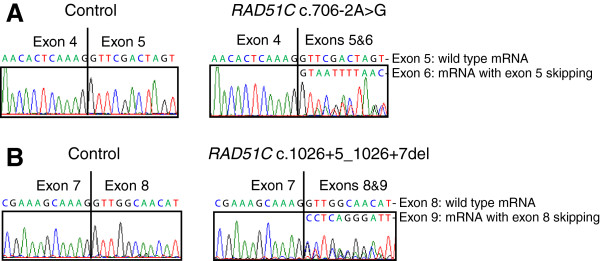
**mRNA analysis for *RAD51C *splicing mutations showing exon skipping. (A)** Electropherograms of Sanger sequencing analysis for a control sample with wild type *RAD51C* mRNA only (left) and for *RAD51C* c.706-2A > G mutation with two types of mRNA: wild type mRNA and mRNA with exon 5 skipping (right). **(B)** Electropherograms of Sanger sequencing analysis for a control sample with wild type *RAD51C* mRNA only (left) and for *RAD51C* c.1026 + 5_1026 + 7del mutation with two types of mRNA: wild type mRNA and mRNA with exon 8 skipping (right).

**Figure 2:**
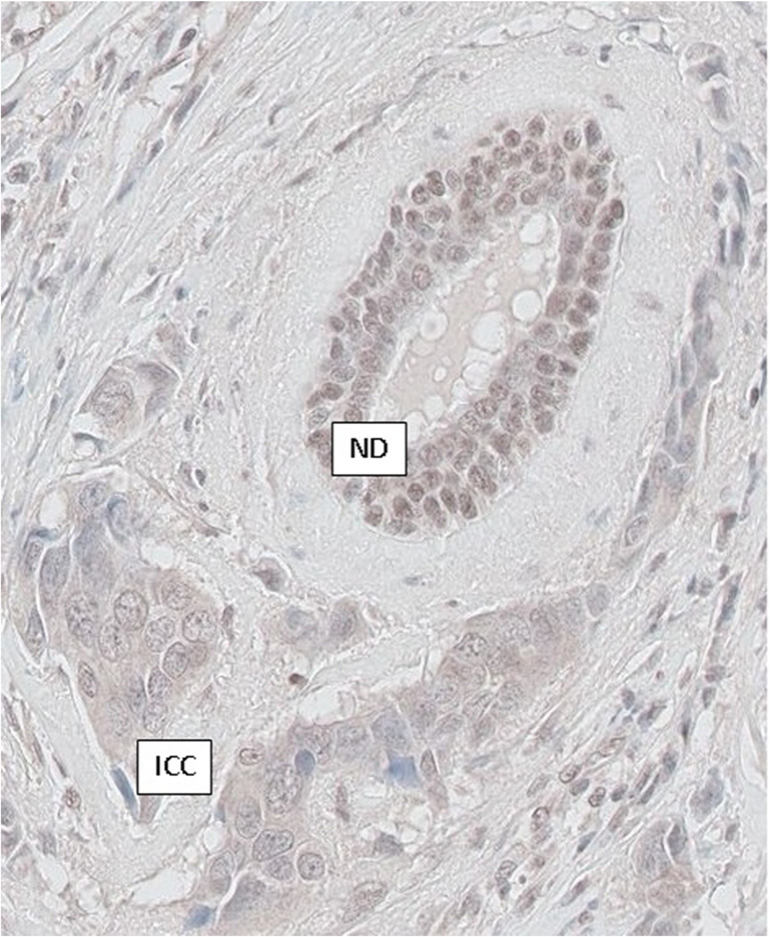
**RAD51B immunohistochemistry in breast tissue of patient carrying the *RAD51B *c.452 + 3A > G mutation.** A brown staining of moderate intensity is observed in the nucleus of non tumor epithelial cells located in normal duct (ND) of the breast tissue. In comparison, no significant staining is detected in the nucleus of invasive carcinoma cells (ICC).

All variants detected on DNA were tested by *in silico* splicing effect prediction according to a previously published pipeline [30]: a greater than 15% decrease of the MaxEntScan score for donor/acceptor splice sites and a greater than 5% decrease of the SpliceSiteFinder-like score for donor/acceptor splice sites were considered to be significant with 96% sensitivity and 83% specificity. Three variants were likely to alter splicing according to this pipeline: *RAD51C* c.706-2A > G and c.1026 + 5_1026 + 7del, and *RAD51B* c.452 + 3A > G. Exon skipping was confirmed by mRNA analysis for the two *RAD51C* mutations (Figure 1). No RNA was available to study the impact of the *RAD51B* c.452 + 3A > G mutation but, using immunohistochemistry with anti-RAD51B antibody, loss of expression of RAD51B protein was observed in breast carcinoma cells from the patient bearing this mutation, as compared with that detected in the nucleus of normal duct cells (Figure 2).

Likely deleterious missense variants reported in this study in the *RAD51B* and *XRCC3* genes are located in the ATP-binding domain of the proteins and result in the replacement of highly conserved amino acids with subsequent high Grantham score (Table 2).

**Figure 3:**
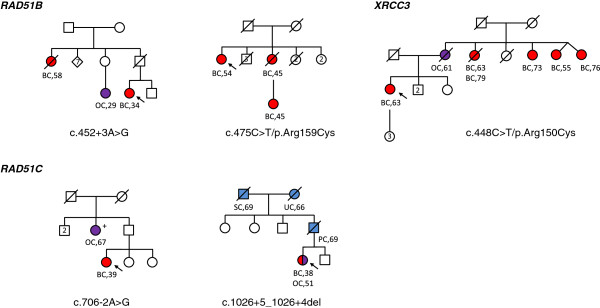
**Pedigrees for *RAD51 *paralog mutation and likely causal variant carriers.** Individuals with breast cancer (BC) are shown as red circles, ovarian cancer (OC) as purple circles, and other cancers as blue circles. SC: stomach cancer; UC: uterine cancer; PC: pancreas cancer. Disease and age in years at diagnosis are given underneath the symbol. The index case is indicated with an arrow. No co-segregation studies have yet been performed, except for *RAD51C* c.706-2A > G: tested relative with OC carried the mutation, indicated by (+).

Mutations and likely deleterious variants were detected in families with both breast and ovarian cancers, except for the *RAD51B* p.Arg159Cys variant that occurred in a family with 3 breast cancer cases (Figure 3). The *RAD51C* c.706-2A > G mutation co-segregated with an ovarian cancer at the age of 67 years for a paternal aunt of the index case. No other co-segregation studies have been performed to date.

## Discussion

This study reports *RAD51* paralog analysis in breast and ovarian cancer cases. To our knowledge, this is the first report of a *RAD51B* mutation and evaluation of the contribution of the *XRCC3* gene to breast and ovarian cancer predisposition.

### *RAD51B* gene

We identified a *RAD51B* mutation and a likely deleterious variant in two patients: the *RAD51B* c.452 + 3A > G mutation was detected in a breast and ovarian cancer family case and the *RAD51B* p.Arg159Cys variant was detected in a family with 3 breast cancer cases.

*RAD51B* has been previously evaluated as a candidate gene for breast cancer predisposition but no mutation was detected in a study of 188 multiple breast cancer family cases (Johnson *et al.*[20]). The low frequency of *RAD51B* mutations may account for the differences observed between our results and those reported by Johnson *et al.,* as previously described for *RAD51C*, *RAD51D* and *XRCC2*[16-18]. More generally concerning *RAD51B* involvement in cancer, previous studies have identified chromosomal rearrangements disrupting *RAD51B* in benign tumours, particularly uterine leiomyomas [31,32]. Overall, haploinsufficiency of *RAD51B* was shown to induce genomic instability in human cells, suggesting its involvement in cancer predisposition [33]. In addition, our findings must be interpreted in the context of two genome-wide association studies (GWAS) that identified the minor allele of single nucleotide polymorphisms (SNPs) in *RAD51B* acting as low risk factors for breast cancer: rs999737 [34] and rs1314913 [35], located in *RAD51B* introns 10 and 7, respectively. Overall, these findings might indicate that *RAD51B* acts as a susceptibility factor or as a major gene depending on the context. Indeed, it cannot be excluded that the minor allele of these SNPs indirectly reflects a major influence of *RAD51B*[36], as a recent study showed that high risk rare mutations can account for some synthetic associations identified by GWAS [37].

*RAD51B* c.452 + 3A > G is a novel mutation. Several arguments strongly support its causality: this variation is absent in the thousands of controls tested in online databases (Exome Variant Server [28], dbSNP [38], 1000 Genomes [29]); *in silico* prediction concluded this variation was likely to result in an out-of-frame exon skipping leading to a truncated or unstable protein; RAD51B immunohistochemistry in breast carcinoma cells of the patient bearing this variation showed a loss of expression of RAD51B.

The *RAD51B* p.Arg159Cys variant is reported in Exome Variant Server: this variant was detected in 2 out of 4,299 controls in European-American populations. We consider this variant to be a likely deleterious variant because it occurs in a functional domain and results in replacement of a highly conserved amino acid with subsequent high Grantham score and Align-Grantham Variation Grantham Deviation (Align-GVGD) maximum score (C65), and very low frequencies are reported in Exome Variant Server. Its occurrence in controls could be explained by an incomplete penetrance.

Overall, for the *RAD51B* gene, one truncating mutation and one likely deleterious variant were detected in 2 out of 142 patients selected for enrichment in breast/ovarian cancer cases. In online databases, one truncating mutation and two likely deleterious variants were detected in 4 out of 4,678 controls (Additional file 4: Table S4). Frequency of *RAD51B* variants was significantly higher in cases (p = 0.012), which suggests *RAD51B* variants are associated with breast/ovarian cancer risks.

### *XRCC3* gene

The *XRCC3* p.Arg150Cys variant was detected in a family with 1 ovarian cancer and 5 breast cancer cases. Like the *RAD51B* missense variant reported in this study, the *XRCC3* p.Arg150Cys variant is reported with a very low frequency in Exome Variant Server (1 out of 4,276 controls in European-American populations). We consider this variant to be a likely deleterious variant because it occurs in a functional domain and results in replacement of a highly conserved amino acid with subsequent high Grantham score and Align-GVGD maximum score (C65).

This study is the first report of *XRCC3* mutation screening in breast and ovarian cancer predisposition. Numerous association studies have evaluated *XRCC3* SNPs as candidate risk factors for breast cancer, but the results of these studies remain controversial. A recent meta-analysis suggested that the minor allele of *XRCC3* p.Thr241Met SNP was a low risk factor and *XRCC3* IVS5-14A > G SNP a low protective factor for breast cancer [39].

### *RAD51C* gene

Several studies have reported *RAD51C* causal mutations in breast and ovarian cancer predisposition [16,40,41]. Two novel *RAD51C* splicing mutations are reported in this study: *RAD51C* c.1026 + 5_1026 + 7del mutation is truncating, resulting in an out-of-frame exon 8 skipping and *RAD51C* c.706-2A > G mutation leads to the loss of 44 amino acids in a functional domain of the protein by an in-frame exon 5 skipping. These two *RAD51C* mutations were detected in families with both breast and ovarian cancer cases, which is consistent with previous studies. As this set of patients was enriched with ovarian cancer cases and due to the low frequency of *RAD51C* mutations, other studies must be conducted in larger series to evaluate whether *RAD51C* confers predisposition to ovarian cancer alone or to both breast and ovarian cancer, which remains controversial [42].

## Conclusions

This study identified the first *RAD51B* mutation in a breast and ovarian cancer family and confirmed that *RAD51* paralog mutations confer breast and ovarian cancer predisposition and are rare events. Identification of families with mutations in the *RAD51B, RAD51C* or *XRCC3* genes and genetic testing of family members could be used to estimate the associated breast and ovarian cancer risks. In view of the low frequency of *RAD51* paralog mutations, international collaboration of family cancer clinics will be required to more accurately estimate their penetrance and establish clinical guidelines. Such studies would be facilitated by the development of Next Generation Sequencing allowing laboratories to simultaneously analyse numerous genes.

## Abbreviations

EMMA: Enhanced mismatch mutation analysis; Align-GVGD: Align-Grantham variation Grantham deviation; GWAS: Genome-wide association studies; SNP: Single nucleotide polymorphism; ND: Normal duct; ICC: Invasive carcinoma cells; BC: Breast cancer; OC: Ovarian cancer; SC: Stomach cancer; UC: Uterine cancer; PC: Pancreas cancer.

## Competing interests

The authors declare that they have no competing interests.

## Authors’ contribution

LG evaluated and interpreted the data, and wrote the paper. VM was involved in design of mutation analysis by EMMA and Sanger sequencing, data analysis and interpretation. GD, EA, BP, CT, DM, CB and CDD performed part of the mutation analysis and interpretation of the data. LC analysed and evaluated part of the data of mutation analysis. AN and XSG were involved in design, evaluation and interpretation of the data of RAD51B immunohistochemistry. MHS, CH and DSL were involved in conception and design of the study, data interpretation and writing the paper. All authors have critically revised and approved the submitted paper.

## Pre-publication history

The pre-publication history for this paper can be accessed here:

http://www.biomedcentral.com/1471-2407/13/484/prepub

## Supplementary Material

Additional file 1: Table S1 Multiplex PCR mixes. Click here for file

Additional file 2: Table S2 Primers. Click here for file

Additional file 3: Table S3 All variants and polymorphisms detected in this study. Click here for file

Additional file 4: Table S4 *RAD51B* variants in cases and controls. Click here for file
