# Supplementary material for: Germline mutation in the RAD51B gene confers predisposition to breast cancer
Source: BMC Cancer. 2013 Oct 19;13:484. doi: 10.1186/1471-2407-13-484 (PMC4016303; doi:10.1186/1471-2407-13-484)
Supplement: Additional file 1: Table S1 — Multiplex PCR mixes. [file 1471-2407-13-484-S1.docx]

Additional file 1: Table S1 Multiplex PCR mixes.

| Multiplex | Amplicon 1  (Length [bp]) | Amplicon 2  (Length [bp]) | Amplicon 3  (Length [bp]) | Amplicon 4  (Length [bp]) | Amplicon 5  (Length [bp]) |
| --- | --- | --- | --- | --- | --- |
| 1 | AFM8 (266) | XRCC2-2 (339) | XRCC2-3c (385) | XRCC3-8 (469) | RAD51D-8+9 (538) |
| 2 | AFM8 (266) | RAD51D-2 (336) | RAD51B-4 (389) | XRCC3-5 (424) | XRCC3-7 (523) |
| 3 | AFM8 (266) | RAD51B-8 (349) | RAD51B-6 (414) | XRCC3-4 (460) | RAD51D-5 (538) |
| 4 | AFM8 (266) | RAD51B-10 (322) | XRCC2-3b (389) | RAD51D-4 (462) | RAD51D-10 (503) |
| 5 | AFM8 (266) | RAD51D-7 (377) | RAD51B-2 (420) | RAD51B-12 (467) | XRCC3-10 (516) |
| 6 | AFM8 (266) | XRCC3-9 (369) | RAD51B-13 (452) | XRCC2-3a (550) |  |
| 7 | AFM8 (266) | RAD51D-11b (370) | RAD51D-3 (396) | RAD51B-9 (468) |  |
| 8 | AFM8 (266) | RAD51D-6 (430) | RAD51B-3 (500) |  |  |
| 9 | AFM8b (263) | RAD51D-1b (315) | XRCC3-6 (485) | RAD51B-7 (538) |  |
| 10 | AFM8b (263) | XRCC2-1a (318) | RAD51D-1a (453) |  |  |
| 11 | AFM8b (263) | XRCC2-1b (429) |  |  |  |
| 12 | AFM8 (266) | RAD51C-5 (421) | RAD51C-7 (475) | RAD51C-2 (548) |  |
| 13 | AFM8 (266) | RAD51C-6 (396) | RAD51C-3 (467) | RAD51C-4 (545) |  |
| 14 | AFM8 (266) | RAD51C-8 (379) | RAD51C-1 (465) | RAD51C-9 (491) |  |

bp: base pairs. AFM8 and AFM8b are control amplicons. Exon numbers are indicated after gene names. Letters a, b, c are used when several amplicons were analysed for the same exon.
