# Supplementary material for: Germline mutation in the RAD51B gene confers predisposition to breast cancer
Source: BMC Cancer. 2013 Oct 19;13:484. doi: 10.1186/1471-2407-13-484 (PMC4016303; doi:10.1186/1471-2407-13-484)
Supplement: Additional file 2: Table S2 — Primers. [file 1471-2407-13-484-S2.docx]

Additional file 2: Table S2 Primers.

| Exon | Forward primer | Reverse primer |
| --- | --- | --- |
| RAD51B-2 | AAGGTTCTATAGCATTCCTTTATCAG | CACCCAAGGAGGCTTGATATT |
| RAD51B-3 | GGATTGGATGCTGGTGCTAT | AAACTCAGTATTTTTGAGGTACTCATC |
| RAD51B-4 | CAAATAAATAATAGAAGGGAAAAGGA | GAGTGGAGAACCATATCCTTGAA |
| RAD51B-5* | ATTCAGATGCAGAGGGGAGA | GAGGTGGAGATTGCCATGAG |
| RAD51B-6 | GCCATTTTGCTTATACCTGCTC | CAAGAAAGACTTCAACCAAAAGG |
| RAD51B-7 | GAAACAGTTTGCTCTGTTTCCA | GATGATGGCGATGTTTGCTA |
| RAD51B-8 | TTGCTACCTGTGTATTTATCAGTCTTC | CTCCGGCTGGGTTCACTG |
| RAD51B-9 | TCACCAAGGCTGAAGGAAAC | GTTGGGACAAGCAGAAATGC |
| RAD51B-10 | AAGCCTTGTGACTTACAGTCCT | GCCAATGCATCTTGGTTTTT |
| RAD51B-11* | GTGCTTGCTTCTGTGGATGA | CACCTTCCCTGCTAGGTCTG |
| RAD51B-12 | TCTCCATGTCCCCAGTTCTC | TTGGGCTTGTTCTTGATCCT |
| RAD51B-13 | TTCCTATGCCATACCCCTTG | ATTGCCCCCTTTCTCATTCT |
| RAD51C-1 | CGGAATGGTGCATAAGTGTG | GAGGAGCTCTTTCACGCTGT |
| RAD51C-2 | TGTTTCTCCACTCCTAGCATCA | CAAGAAGGGATAATGAAGTAACACAA |
| RAD51C-3 | TCTGTTGCCTTGGGGAGTAT | GGCTGTGGCATTTCTCATTT |
| RAD51C-4 | GCGACGGATAGCATTAGGAG | TCTCAATTGGCTTTGACTTTGA |
| RAD51C-5 | CAGGCATTGGGGATGATATAG | TGGAAACCAACCAAACGTAAC |
| RAD51C-6 | CATGCCACCATGTCTGGTTA | CTGTGTCTGGCCACTCAATAAA |
| RAD51C-7 | GCTTGATGATATGGAAAATTGACA | GGTGATATCAGACAAGGCAACA |
| RAD51C-8 | ACGGGTAATTTGAAGGGTGT | TGGGGACAATGTTCTAAGCA |
| RAD51C-9 | CGCCTGGCCCTAGAATAAA | GGCCACATGAGATCAGCTTT |
| RAD51D-1a | GCAGAAGGTAGAGGCCGAAT | GTAAGGCCAGGGCACAGTC |
| RAD51D-1b | GCGCCTGTGTCCTCTCTAGG | CGGCCCTCTAGGAATGGAG |
| RAD51D-2 | ATTTCACACTGGCCTCCTGA | TCCTGACTTCTGACTCCAAGTG |
| RAD51D-3 | AACAACAATAACAAAAGTCCATCC | ATGTCCTGACCCCTTTCCTT |
| RAD51D-4 | TTGGGTGTAGGGGAAAATCA | GCCAGGACAGTCTCGATTTC |
| RAD51D-5 | ACCAGAGAAGGCACATTTGG | CCACATTTGCTGCCATACAG |
| RAD51D-6 | GCCCAACAGAGAGAAGATGG | ACAGGGGTGGCTTAAGGAAT |
| RAD51D-7 | CAGCCACCACTGTGACAACT | GCAAGCATAGTTGGAGTCACC |
| RAD51D-8+9 | GCTTGCTGTATTTGGGATGG | CCAGGGAAGCTGGGATATGT |
| RAD51D-10 | GGAGGCCCAGGTTCTAGTTC | GAAACCTGTTGGCTGGAAGA |
| RAD51D-11 | GATTCTCCTGGACACCATCG | ACAGAGAGTGAGGCCAAGGA |
| XRCC2-1a | CAAGAAAAGCACCAGCTGTC | AGGTGAGAGGTGGCAGAGG |
| XRCC2-1b | AGGCCCGCACACCCTATT | CTCCCACCTCGGAGGACT |
| XRCC2-2 | CCACAGCACCCAGCCTAA | CAACCTGTGTCTCATTTTCCAA |
| XRCC2-3a | CTTCCAACCTTCCCACCAG | GCGGTCTATCCAGTAAAAAGC |
| XRCC2-3b | CTGCAGTAGTAGCACCCACTT | TTTGAGAATCATCTTGTTTGGAGA |
| XRCC2-3c | GAACCTTCTCATGCCTCTCG | AGGAGGCACACATAGGAGGA |
| XRCC3-4 | CTGCTGTCACTTCCCTGGTT | CGCCCTCGGTTTAATAATCA |
| XRCC3-5 | GTCCCTACCCACCTTGTCC | AAGCAAGATGGGAACTCTGG |
| XRCC3-6 | CTTGCTCACCCCCATGAC | AGGGTGAGAAGGAGGGAGAC |
| XRCC3-7 | GAGGGTGAGAAAAAGCACCA | CCTGCCAGTTCCTCTCAGTC |
| XRCC3-8 | GCACTGTGAGCCCCAGGT | GGTTAGGCACAGGCTGCTAC |
| XRCC3-9 | CTGTGCATCAACCAGGTGAG | CCCAGAACCTGAGAAACAGG |
| XRCC3-10 | TGTGCTCTTCCAGGTGACAG | GGAAGAGCTGTGTCTGAACCA |

Exon numbers are indicated after gene names. Letters a, b, c are used when several amplicons were analysed for the same exon. Primers were used for EMMA and sequencing analysis except for the two amplicons indicated by (*) that were used for direct sequencing.
