# Supplementary material for: Germline mutation in the RAD51B gene confers predisposition to breast cancer
Source: BMC Cancer. 2013 Oct 19;13:484. doi: 10.1186/1471-2407-13-484 (PMC4016303; doi:10.1186/1471-2407-13-484)
Supplement: Additional file 3: Table S3 — All variants and polymorphisms detected in this study. [file 1471-2407-13-484-S3.docx]

Additional file 3: Table S3 All variants and polymorphisms detected in this study.

| Gene | Region | Nucleotide nomenclature | Protein nomenclature | dbSNP reference |
| --- | --- | --- | --- | --- |
| *RAD51B* | Intron 2 | c.85-218T>A |  | rs79415557 |
|  | Exon 5 | c. 359 T>C | p.Met120Thr | rs142567687 |
|  | Intron 5 | c.452+3A>G |  | ND |
|  | Exon 6 | c.475C>T | p.Arg159Cys | rs61755649 |
|  | Exon 6 | c.539A>G | p.Tyr180Cys | rs28910275 |
|  | Exon 7 | c.619G>T | p.Val207Leu | rs28908168 |
|  | Exon 7 | c.728A>G | p.Lys243Arg | rs34594234 |
|  | Intron 7 | c.757-49T>C |  | rs34573256 |
|  | Intron 7 | c.757-26T>C |  | rs10129646 |
|  | Intron 8 | c.854-113C>G |  | rs45497193 |
|  | Intron 9 | c.957+90A>G |  | ND |
|  | Intron 10 | c.1037-8T>C |  | rs181459801 |
| *RAD51C* | 5’UTR | c.-42-40G>A |  | ND |
|  | Exon 1 | c.106G>A | p.Glu36Lys | ND |
|  | Exon 2 | c.376G>A | p.Ala126Thr | rs61758784 |
|  | Intron 3 | c.572-149T>C |  | ND |
|  | Intron 3 | c.572-85T>G |  | ND |
|  | Intron 3 | c.572-17G>T |  | ND |
|  | Intron 4 | c.706-2A>G |  | ND |
|  | Exon 5 | c.801G>A | p.Gln267Gln | ND |
|  | Intron 7 | c.965+25C>G |  | ND |
|  | Intron 8 | c.1026+5_1026+7del |  | ND |
| *RAD51D* | Exon 1 | c.26G>C | p.Cys9Ser | rs140825795 |
|  | Exon 3 | c.211C>T | p.Leu71Phe | ND |
|  | Intron 4 | c.264-50_264-49del |  | ND |
|  | Exon 4 | c.292G>A | p.Asp98Asn | ND |
|  | Exon 8 | c.620C>T | p.Ser207Leu | ND |
|  | Intron 9 | c.738+54T>C |  | ND |
|  | Intron 10 | c.903+53C>T |  | rs45496096 |
| *XRCC2* | 5'UTR | c.-220G>A |  | ND |
|  | 5'UTR | c.-213G>A |  | ND |
|  | 5'UTR | c.-126C>A |  | ND |
|  | 5'UTR | c.-76C>T |  | ND |
|  | Intron 1 | c.40-10C>T |  | rs3218472 |
|  | Exon 3 | c.563G>A | p.Arg188His | rs3218536 |
|  | 3'UTR | c.*35C>T |  | rs140280051 |
| *XRCC3* | Intron 4 | c.55+95_55+96dup |  | ND |
|  | Intron 4 | c.56-35dup |  | rs3212044 |
|  | Intron 5 | c.193+133C>A |  | ND |
|  | intron 6 | c.407-83G>A |  | ND |
|  | Exon 7 | c.441G>A | p.Pro147Pro | rs138987760 |
|  | Exon 7 | c.448C>T | p.Arg150Cys | rs150729160 |
|  | Exon 7 | c.555C>T | p.Ala185Ala | ND |
|  | Intron 7 | c.561+75G>A |  | ND |
|  | Intron 8 | c.775-38C>T |  | ND |
|  | Intron 9 | c.821+14T>C |  | ND |
|  | Intron 9 | c.821+23_821+24del |  | ND |
|  | Intron 9 | c.822-57C>T |  | rs17101777 |

All variants were unique except for *RAD51B* c.728A>G, *RAD51B* c.854-113C>G, *RAD51C* c.106G>A and *XRCC3* c.822-57C>T detected in 2 patients and *XRCC3* c.193+133C>A detected in 3 patients. NCBI GenBank references used for variant annotation are NM_133509.3, NM_058216.1, NM_002878.3, NM_005431.1, NM_005432.3 for *RAD51B*, *RAD51C*, *RAD51D*, *XRCC2* and *XRCC3*, respectively, except for variants *RAD51B* c.1037-8T>C (NM_133510.3) and *RAD51D* c.292G>A (NM_001142571.1). UTR: untranslated region. ND: not described in dbSNP.
