# Supplementary material for: Germline mutation in the RAD51B gene confers predisposition to breast cancer
Source: BMC Cancer. 2013 Oct 19;13:484. doi: 10.1186/1471-2407-13-484 (PMC4016303; doi:10.1186/1471-2407-13-484)
Supplement: Additional file 4: Table S4 — RAD51B variants in cases and controls. [file 1471-2407-13-484-S4.docx]

**Additional file 4: Table S4 *RAD51B* variants in cases and controls.**

| Genetic variation | Protein alteration | rs number | Align-GVGD* | SIFT^§^ | PolyPhen-2 | Cases (%) | Controls (%) | Population |
| --- | --- | --- | --- | --- | --- | --- | --- | --- |
| c.428C>T | p.Thr143Ile | rs143457995 | C65 | 0.00 | Probably damaging | 0/142 (0.00) | 1/4300 (0.0002) | European-Americans^†^ |
| c.139C>T | p.Arg47X | rs200355697 | - | - | - | 0/142 (0.00) | 1/379 (0.2639) | 1000 Genomes Europeans^‡^ |
| c.475C>T | p.Arg159Cys | rs61755649 | C65 | 0.00 | Probably damaging | 1/142 (0.70) | 2/4299 (0.0465) | European-Americans^†^ |
| c.452+3A>G | Unstable or truncated protein | - | - | - | - | 1/142 (0.70) | ND |  |

* Align-Grantham Variation Grantham Deviation (Align-GVGD) classes range from C0 to C65; C65 class variants are the most likely to interfere with protein function [22, 23].

§ Normalised probabilities smaller than 0.05 are predicted to be deleterious [24, 25].

† European-Americans from Exome Variant Server [28].

‡ European controls from 1000 Genomes project [29].

ND: not described in the literature and in Exome Variant Server, 1000 Genomes, or dbSNP [38].

Frequencies of *RAD51B* variants between controls from Exome Variant Server (European-Americans) and 1000 Genomes project (Europeans) were not significantly different (p=0.287). Frequency of *RAD51B* variants was significantly higher in cases vs the two control samples (p=0.012).
